# Myocardial Mitochondrial DNA Drives Macrophage Inflammatory Response through STING Signaling in Coxsackievirus B3-Induced Viral Myocarditis

**DOI:** 10.3390/cells12212555

**Published:** 2023-10-31

**Authors:** Andong Qin, Zhenke Wen, Sidong Xiong

**Affiliations:** Jiangsu Key Laboratory of Infection and Immunity, Institutes of Biology and Medical Sciences, Soochow University, Suzhou 215123, China

**Keywords:** CVB3, VMC, mtDNA, STING, macrophage inflammatory response

## Abstract

Coxsackievirus B3 (CVB3), a single-stranded positive RNA virus, primarily infects cardiac myocytes and is a major causative pathogen for viral myocarditis (VMC), driving cardiac inflammation and organ dysfunction. However, whether and how myocardial damage is involved in CVB3-induced VMC remains unclear. Herein, we demonstrate that the CVB3 infection of cardiac myocytes results in the release of mitochondrial DNA (mtDNA), which functions as an important driver of cardiac macrophage inflammation through the stimulator of interferon genes (STING) dependent mechanism. More specifically, the CVB3 infection of cardiac myocytes promotes the accumulation of extracellular mtDNA. Such myocardial mtDNA is indispensable for CVB3-infected myocytes in that it induces a macrophage inflammatory response. Mechanistically, a CVB3 infection upregulates the expression of the classical DNA sensor STING, which is predominantly localized within cardiac macrophages in VMC murine models. Myocardial mtDNA efficiently triggers STING signaling in those macrophages, resulting in strong NF-kB activation when inducing the inflammatory response. Accordingly, *STING*-deficient mice are able to resist CVB3-induced cardiac inflammation, exhibiting minimal inflammation with regard to their functional cardiac capacities, and they exhibit higher survival rates. Moreover, our findings pinpoint myocardial mtDNA as a central element driving the cardiac inflammation of CVB3-induced VMC, and we consider the DNA sensor, STING, to be a promising therapeutic target for protecting against RNA viral infections.

## 1. Introduction

Viral myocarditis (VMC) is a cardiac inflammatory disorder that is caused by a viral infection which can further progress into chronic myocarditis, dilated cardiomyopathy (DCM), and ultimately, heart failure [1,2,3]. VMC is a relatively common disease and a leading cause of sudden cardiac death among adolescents and young adults [4,5,6]. Multiple viruses are associated with VMC, with coxsackievirus B3 (CVB3) being the most common pathogen [7]. CVB3 belongs to the picornavirus family of the enterovirus genus, and it is a positive-sense, single-stranded RNA virus [8]. However, due to the lack of knowledge regarding the pathological mechanisms underlying CVB3-induced VMC, targeted therapeutic options are extremely limited in clinical practice settings.

Since CVB3 primarily infects cardiac myocytes, the potential role of myocardial damage in VMC pathogenesis has become an active area of investigation. The innate immune system provides the first line of defense against invading pathogens, and it plays a vital role in viral pathogenesis. Upon CVB3 infection in the acute phase of VMC, cardiac tissue is mainly infiltrated by immune cells such as macrophages, dendritic cells (DCs), and neutrophils. The pathogen-associated molecular patterns (PPARs) of CVB3 can be recognized with pathogen recognition receptors (PRRs), such as Toll-like receptors (TLRs), resulting in the activation of the nuclear factor kappa B (NF-κB) signal pathway [9,10,11]. Activated NF-κB leads to the translocation of its p50 and p65 subunits from the cytoplasm to the nucleus, and it drives the expression of proinflammatory cytokines, including tumor necrosis factor α (TNF-α) and interleukin 6 (IL-6) [12,13]. Ultimately, CVB3 can result in direct injury to cardiac cells by inducing an inflammatory response, which serves as the major immune pathogenesis of CVB3-induced VMC [7]. Therefore, elucidating the molecular mechanisms that control the regulation of cardiac inflammation could provide a novel therapeutic strategy for CVB3-induced VMC.

The stimulator of interferon genes (STING), also known as Transmembrane protein 173 (TMEM173), MPYS, MITA, or ERIS, is a critical inflammatory molecule involved in DNA sensing. Under stimulated conditions, activated STING can recruit TANK-binding kinase 1 (TBK1) and trigger the activation of downstream signaling, such as interferon (IFN) and NF-κB [14,15,16,17]. Accordingly, STING has been shown to be involved in the immune pathogenesis of multiple inflammatory disorders such as acute pancreatitis, non-alcoholic steatohepatitis (NASH), and inflammatory lung disease [18,19,20,21]. Of note, STING might also be relevant in the inflammatory pathogenesis of cardiovascular disorders [22]. However, whether and how STING is involved in CVB3-induced VMC remains largely unknown.

In this study, we aimed to explore the pathophysiological role of myocardial damage in the cardiac inflammatory response of CVB3-induced VMC, and we identified STING as the critical mediator for aggravating the macrophage inflammatory response. We uncovered myocardial mitochondrial DNA (mtDNA) as an essential component in driving cardiac macrophage inflammation, shedding new light on the molecular mechanisms underpinning the immune pathogenesis of CVB3-induced VMC.

## 2. Materials and Methods

### 2.1. Mice and VMC Model

Five-week-old wild-type (WT) C57BL/6J mice were purchased from Shanghai SLAC Laboratory Animal Co., Ltd. (SLAC, Shanghai, China). *STING*-deficient mice (B6(Cg)-*Sting^tm1.2Camb^*/J, Stock No:025805) were purchased from the Jackson Laboratory (Bar Harbor, ME, USA). CVB3 was passaged in HeLa cells, and mice were intraperitoneally infected with 10^5^ TCID50 CVB3 (Nancy strain) to induce the VMC model. All mice were housed in a specific pathogen-free facility in the animal breeding center of Soochow University. All animal experiments were performed in accordance with the Guide for the Care and Use of Laboratory Animals (National Academy of Science, 2011), and they were approved by the Ethical Committee of Soochow University (Suzhou, China).

### 2.2. Cell Isolation, Preparation, and Culture

Bone marrow-derived macrophages (BMDMs) were prepared as previously described [23]. To differentiate the BMDMs, freshly isolated cells were cultured in the presence of M-CSF (50 ng/mL), 10% FBS, 2 mM L-glutamine, 100 U/mL penicillin, and 100 μg/mL streptomycin for 6 days. BMDMs were harvested on day 6 for the proceeding experiments.

Cardiomyocytes were isolated from 48 h old neonatal mice, or CVB3-infected mice, as previously described [24]. Mouse cardiac tissues were cut into 1 mm^3^ in a D-Hanks balanced salt solution (HBSS). Cardiomyocytes were dissociated using HBSS containing 25 μg/mL LiberaseTM TH (SKU:5401110001, Roche Diagnostics, GmbH, Mannheim, Germany), at 37 °C, and they were agitated every 5 min until the tissue was completely dissociated. Cells were collected from the supernatant via centrifugation at 200× *g* for 5 min. Then, the cells were plated in 60 mm plastic Petri dishes at 37 °C, for 2 h, to separate cardiomyocytes from fibroblasts via differential plating.

Mouse cardiac macrophages were isolated using FACS, as previously described [25]. The cardiac tissues were cut into 1 mm^3^ pieces, digested using 25 μg/mL LiberaseTM TH (SKU:5401110001, Roche, Diagnostics, GmbH, Mannheim, Germany) in HBSS, at 37 °C, for 15 min, and filtered using a 40 μm cell strainer (BD Falcon, Franklin Lakes, NJ, USA). The remaining tissues were digested using a fresh digestion solution, and they were similarly filtered. The suspension obtained was combined and subjected to erythrocyte depletion using a Red Cell Lysis Buffer (Cat#C3702, Beyotime Biotechnology, Shanghai, China), in accordance with the manufacturer’s protocol. The collected cells were re-suspended in the FACS buffer, labeled with a PE-conjugated anti-F4/80 antibody (Cat#565410, BD Pharmingen, Franklin Lakes, NJ, USA), and subjected to cell sorting using FACS Aria II (BD Biosciences, San Jose, CA, USA).

### 2.3. Mitochondrial DNA Isolation

Mitochondrial DNA was extracted using a commercial Mitochondrial DNA isolation kit (Cat#ab65321, Abcam, Cambridge, MA, USA). The experimental process was conducted in accordance with the experimental standard procedures provided with the product.

### 2.4. Echocardiography

The echocardiographic examination of mice was performed as previously described [26,27]. To assess cardiac function, mice were anesthetized with 0.5–1.0% isoflurane and placed on a heating pad to maintain body temperature. Echocardiographic parameters were measured using a high-resolution ultrasound imaging system with a 40-MHz linear transducer (Vevo 2100, Visual Sonics, Toronto, ON, Canada).

### 2.5. Quantitative Polymerase Chain Reaction

Total RNA was isolated using the RNAiso Plus total RNA extraction reagent (Cat#9108, Takara, Dalian, China), in accordance with the manufacturer’s instructions. Then, the RNA (500 ng) was used to generate cDNA via the PrimeScriptTM 1st Strand cDNA Synthesis Kit (Cat#6110A, Takara, Dalian, China), and qRT-PCR was performed using PowerUp™ SYBR™ Green (Cat#a25742, Applied Biosystems, Carlsbad, CA, USA) in the QuantStudio-6 system (Applied Biosystems, Carlsbad, CA, USA). The primers used are as follows: *STING* (F: GCCTTCAGAGCTTGACTCCA, R: CCCTGGTAAGATCAACCGCA), *TNF-α* (F: CATCTTCTCAAAATTCGAGTGACAA, R: TGGGAGTAGACAAGGTACAACCC), *IL-6* (F: GGAAATCGTGGAAATGAG, R: AGGACTCTGGCTTTGTCT), *MCP-1* (F: TTCCCTTGCCTGGTCCCT, R: GTTTTCCCCCAGCCAGCT), *CVB3* (F: ATCAAGTTGCGTGCTGTG, R: TGCGAAATGAAAGGAGTGT), and *GAPDH* (F: AGCAGTCCCGTACACTGGCAAAC, R: TCTGTGGTGATGTAAATGTCCTCT). The reverse primer for CVB3 Positive-strand was: CACCGGATGGCCAATCCCA, and for Negative-strand was: GCGAAGAGTCTATTGAGCTA. The average threshold cycle of quadruplicate reactions was employed for all subsequent calculations using the 2^−ΔΔCt^ method. Gene expression was normalized to *GAPDH*. The data shown are the average from at least three independent experiments, with two technical replicates per experiment. Mean ΔCt values were used to evaluate the expression of the mitochondrial gene and nuclear gene. The primers used are as follows: *ND1*(F: CTAGCAGAAACAAACCGGGC, R: CCGGCTGCGTATTCTACGTT), *16SrRNA* (F: CCGCAAGGGAAAGATGAAAGAC. R: TCGTTTGGTTTCGGGGTTTC), and *HK2* (F: GCCAGCCTCTCCTGATTTTAGTGT, R: GGGAACACAAAAGACCTCTTCTGG).

### 2.6. Western Blot

The total protein from heart tissues and cells was extracted using a RIPA buffer mixed with phenyl methane sulfonyl fluoride (PMSF). Extracted proteins were separated with 10% SDS-PAGE and transferred to a PVDF film (Millipore, Bedford, MA, USA). The PVDF film was blocking with 5% milk, and then was incubated with the primary antibody overnight at 4 °C. The primary antibodies used are as follows: STING Rabbit mAb (Cat#13647S, Cell Signaling Technology, Danvers, MA, USA), cGAS Rabbit mAb (Cat#31659S, Cell Signaling Technology, Danvers, MA, USA), TBK1/NAK Rabbit mAb (Cat#3504S, Cell Signaling Technology, Danvers, MA, USA), Phospho-TBK1/NAK Rabbit mAb (Cat#5483S, Cell Signaling Technology, Danvers, MA, USA), NF-κB/p65 Rabbit mAb (Cat#8242S, Cell Signaling Technology, Danvers, MA, USA), Phospho-NF-κB /p65 Rabbit mAb (Cat#3033S, Cell Signaling Technology, Danvers, MA, USA), and GAPDH Antibody (Cat#AF0911, Affinity Biosciences, Cincinnati, OH, USA). The HRP-labeled goat anti-rabbit IgG antibody (Cat#4030-05, Southern Biotech, Birmingham, AL, USA) was used as the secondary antibody. The blots were imaged using the Amersham Imager 600 (GE Healthcare, Waukesha, WI, USA) and analyzed with Image J software (version1.50i, Waukesha, WI, USA).

### 2.7. Flow Cytometry

The mouse heart tissue was cut into small pieces, digested with 25 mg/mL Liberase TH (SKU:5401110001, Roche, Diagnostics, GmbH, Mannheim, Germany) for 30 min, filtered through a 70 μm filter, and re-suspended in PBS. Single-cell suspensions were incubated with PerCP-CyTM 5.5 Mouse Anti-Mouse CD45 (Cat#552950, BD Pharmingen, San Diego, CA, USA), PE Rat Anti-Mouse F4/80 (Cat#565410, BD Pharmingen, San Diego, CA, USA), APC Anti-Mouse CD11c (Cat#117310, Biolegend, San Diego, CA, USA), Alexa Fluor^®^ 488 anti-mouse Ly-6G Antibody (Cat#127626, Biolegend, San Diego, CA, USA), APC Rat Anti-Mouse CD8 (Cat#553035, BD Pharmingen, San Diego, CA, USA), and PE Rat Anti-Mouse CD4 (Cat#561832, BD Pharmingen, San Diego, CA, USA). Flow cytometric analysis was conducted using a FACS canto Ⅱ (Becton Dickinson, San Jose, CA, USA). Data were analyzed using FlowJoTM(Version 10, FlowJoTM Software, Ashland, OR, USA).

### 2.8. HE and IHC Staining

Mouse heart tissue was fixed with 10% formalin for at least 24 h, embedded in paraffin, and cut into sections that were 5 μm thick. Hematoxylin and eosin (H and E) staining was used to assess the inflammation level. Pathology scores were assessed as previously described [28]. Immunohistochemical (IHC) staining was performed in accordance with the manufacturer’s instructions, using the rabbit-specific IHC polymer detection kit HRP/DAB (Cat# ab209101, Abcam, Cambridge, UK). Images were captured using a Nikon Eclipse 90i microscope (Nikon, Kawasaki, Japan).

### 2.9. ELISA

The serum and cell culture supernatant levels of TNF-α, IL-6, and MCP-1 were determined using an enzyme-linked immunosorbent assay (ELISA) kit (Cat# 88-7324-88, #88-7064-88, #88-7391-88, Invitrogen, Camarillo, CA, USA). The Serum cTnI level was detected using the High Sensitivity Mouse Cardiac Troponin-I ELISA kit (CTNI-1-HS, Life Diagnostics, West Chester, PA, USA). All ELISA experiments were performed in accordance with the manufacturers’ guidelines.

### 2.10. Immunofluorescence

Cells were fixed with 4% paraformaldehyde (PFA), in PBS, at room temperature (RT) for 20 min; then, they were subsequently blocked and permeabilized in a blocking buffer (2% BSA, 0.2% Triton X-100 in PBS) for 30 min, stained with an anti-DNA antibody (Cat#CBL186, Millipore Sigma, Burlington, MA, USA), and diluted in a blocking buffer, at 4 °C, overnight. After washing with PBS, cells were incubated with DyLight 488 labeled Goat Anti-Rabbit IgG H and L antibody (Cat#ab96899, Abcam, Burlingame, CA, USA) for 30 min, and counterstained with DAPI at room temperature. Images were obtained using a Nikon A1R confocal microscope (Nikon, Kawasaki, Japan).

### 2.11. Statistical Analyses

Data are expressed as mean ± SEM. T-tests were used to compare data between the two groups, as appropriate. A one-way ANOVA with Tukey’s post-hoc test was utilized to test differences between multiple groups. Survival was estimated using a Kaplan–Meier survival curve. Statistical analysis was performed using GraphPad Prism software (version 6.01, GraphPad Software, Inc, La Jolla, CA, USA), and *p* < 0.05 was considered statistically significant.

## 3. Results

### 3.1. CVB3-Infected Cardiomyocytes Release mtDNA to Induce an Inflammatory Response

**Figure 1:**
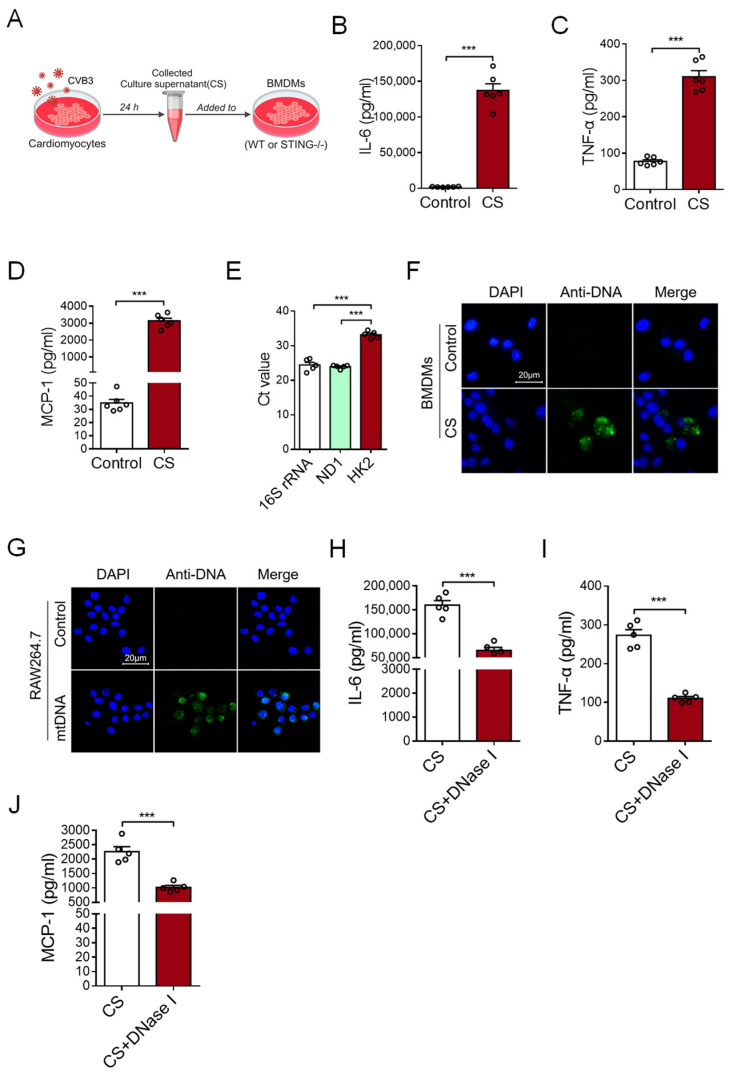
Myocardial mtDNA drives macrophage inflammatory responses. (**A**) Schematic diagram of the experimental design. (**B**–**D**) BMDMs were cultured with a conditioned medium using a culture supernatant (CS) from CVB3-infacted cardiomyocytes, and IL-6, TNF-α, and MCP-1 production was detected with ELISA. Mean ± SEM from six independent experiments in each group. (**E**) Ct values of mitochondrial gene 16S rRNA, ND1, and nuclear gene HK2 in the culture supernatant of CVB3-infected cardiomyocytes. Mean ± SEM from five independent experiments in each group. (**F**) BMDMs were cultured with or without CS from CVB3-infacted cardiomyocytes, and the intracellular DNA (green) content was detected using immunofluorescence. (**G**) RAW264.7 cells were cultured with or without mtDNA from CVB3-infacted cardiomyocytes; then, the intracellular DNA (green) content was detected. Scale bar in (**F**,**G**) 20 μm. (**H**–**J**) CS from CVB3-infected cardiomyocytes were pretreated with or without DNase I, and they were subsequently incubated with BMDMs when analyzing the inflammatory responses. Mean ± SEM from five independent experiments in each group. *** *p* < 0.001 with *t*-test (**B**–**D**,**H**–**J**) and ANOVA (**E**).

To investigate the possible effect of CVB3-infected cardiomyocytes on cardiac inflammation, primary cardiomyocytes were isolated from murine cardiac tissues and infected with CVB3 for 24 h. After that, the culture supernatant (CS) of CVB3-infected cardiomyocytes was tested to find whether it could induce an inflammatory response by stimulating bone marrow-derived macrophages (BMDMs) (Figure 1A). This CS treatment substantially promoted expressions of IL-6, TNF-α, and MCP-1 at both the mRNA and protein levels in BMDMs (Figure 1B–D and Figure S1A), suggesting that CVB3-infected cardiomyocytes play an effective role in inducing cardiac inflammation.

It is well-known that nucleic acids, including DNA and RNA, are crucial for inducing inflammatory responses. Although RNA is much more susceptible to hydrolysis than DNA because of the hydroxyl group, we analyzed the content of DNA in the CS to explore how CVB3-infected cardiomyocytes could drive an inflammatory response. Of note, we uncovered that the levels of mtDNA (*16S rRNA* and *ND1*) were significantly higher than nuclear DNA (*HK2*) in the CS of CVB3-infected cardiomyocytes (Figure 1E), suggesting that CVB3 infection promotes the release of myocardial mtDNA. Furthermore, when the CS from CVB3-infected cardiomyocytes were incubated with BMDMs, the DNA component within BMDMs could be visualized using the anti-DNA antibody (Figure 1F). Additionally, when mtDNA was isolated from CVB3-infected cardiomyocytes and added into the culture of macrophage cell line RAW264.7 cells, intracellular mtDNA could also be readily observed in RAW264.7 cells (Figure 1G).

To confirm the critical role of myocardial mtDNA in inducing an inflammatory response, the CS of CVB3-infected cardiomyocytes was treated with DNase I prior to its subsequent incubation with macrophages. We found that pretreatment with DNase I fundamentally abrogated the effect of CS in terms of its ability to induce an inflammatory response (Figure 1H–J and Figure S1B). Hence, CVB3 infection promotes the release of mtDNA from cardiomyocytes when inducing inflammation.

### 3.2. CVB3 Infection Upregulates STING Expression in Cardiac Macrophages

**Figure 2:**
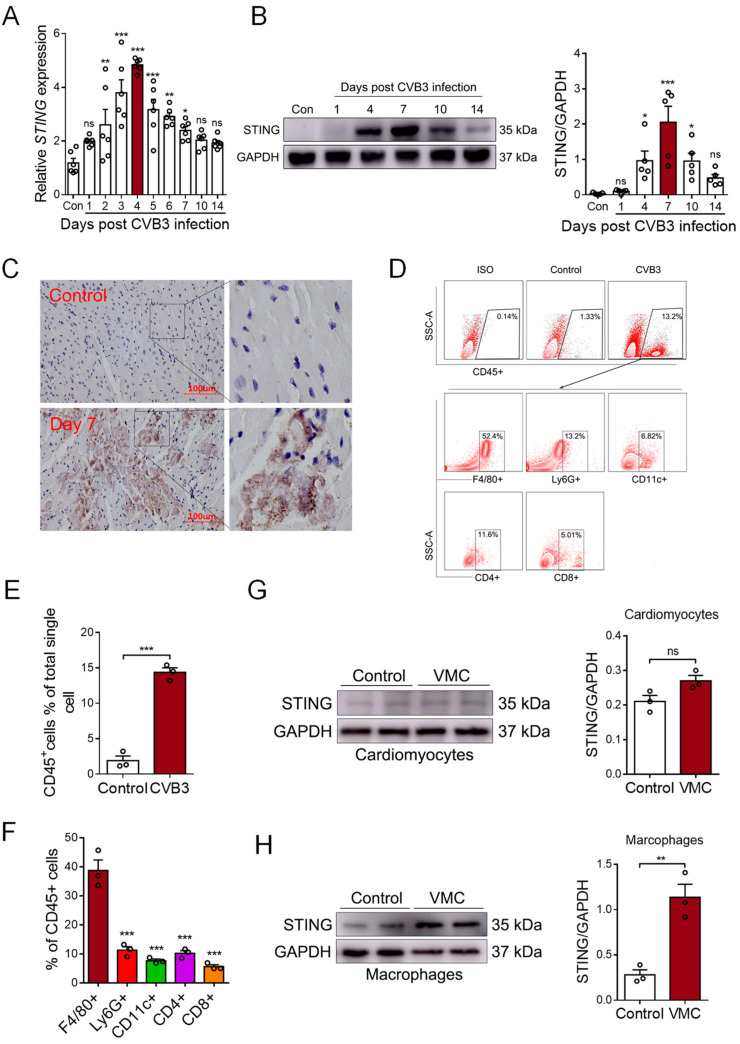
CVB3 infection promotes STING expression in myocardial macrophages. (**A**) Myocardial mRNA levels of STING in CVB3-infected mice were detected using qPCR at the indicated time. Mean ± SEM from six mice in each group. (**B**). Myocardial protein levels of STING in CVB3-infected mice were detected using immunoblots at the indicated time. Mean ± SEM from five mice in each group. (**C**) Immunohistochemical staining for the myocardial STING protein in CVB3-infected mice. (**D**–**F**) Myocardial infiltration of immune cells were characterized using flow cytometry, as indicated. Mean ± SEM from three mice in each group. (**G**,**H**) Cardiomyocytes and macrophages from CVB3-infected mice were assessed in order to find the STING protein using immunoblots. * *p* < 0.05, ** *p* < 0.01, *** *p* < 0.001 with ANOVA (**A**,**B**,**F**) and *t*-test (**E,G**,**H**), ns: no significant difference.

STING is a well-known mediator involved in DNA sensing and pro-inflammatory diseases. To detect whether STING was involved in myocardial mtDNA-induced inflammation in vivo, we analyzed the cardiac expression of STING in CVB3-induced VMC mice. We found that the mRNA level of *STING* gradually increased post-CVB3 infection and it peaked on day 4 (Figure 2A). Moreover, the protein level of STING peaked on day 7, post-CVB3 infection; then, it began to gradually diminish on day 14 (Figure 2B), demonstrating that CVB3 infection efficiently promoted STING expression in the cardiac tissues of VMC mice. Furthermore, immunohistochemistry staining confirmed a significant increase in STING protein in the inflammatory cardiac tissues of VMC mice (Figure 2C), and thus, STING may play a potential role in CVB3-induced cardiac inflammation.

To identify the cellular source of cardiac STING, single-cell suspensions were generated from the cardiac tissues of VMC mice and analyzed using FACS on day 7, post-CVB3 infection. Flow cytometric analyses showed a significant infiltration of CD45^+^ immune cells in the cardiac tissues of VMC mice (Figure 2D,E). The majority of infiltrated immune cells were F4/80^+^ macrophages (Figure 2F). And when isolating cardiomyocytes and cardiac macrophages to detect STING expression, we found that the STING protein mainly increased in macrophages, but not in cardiomyocytes (Figure 2G,H). Thus, although CVB3 could not efficiently infect macrophages [29], cardiac macrophages comprise the major cell subset that expresses STING upon CVB3 infection.

### 3.3. CVB3 Infection Activates Cardiac STING Signaling in the VMC Model

**Figure 3:**
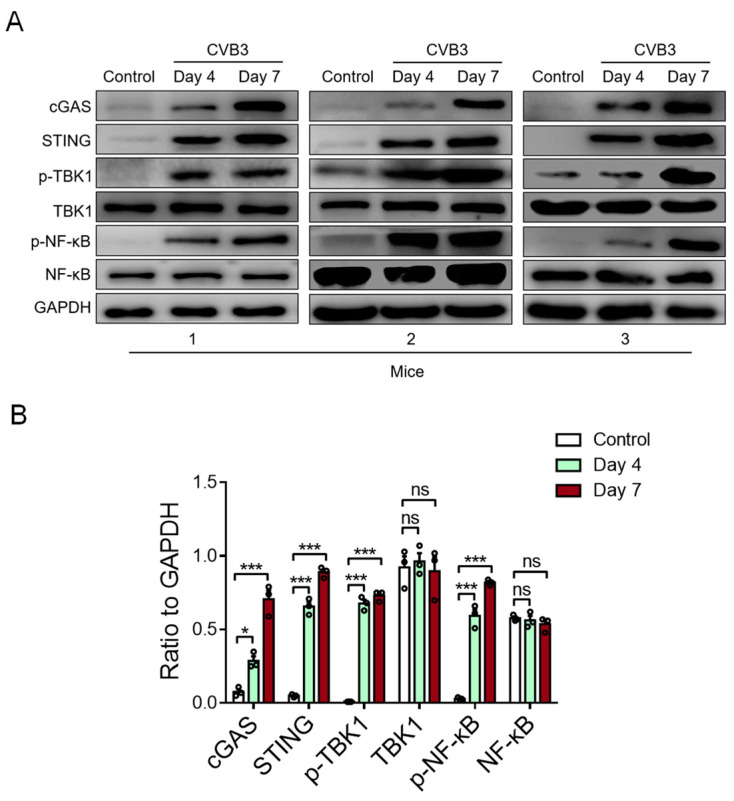
CVB3 infection activates myocardial STING signaling in vivo. The protein expressions of cGAS, STING, p-TBK1, total TBK1, p-NF-kB, and total NF-kB in myocardial tissues from CVB3-infected mice were detected using immunoblots at the indicated time. Representative (**A**) and mean ± SEM (**B**) from three mice in each group. * *p* < 0.05, *** *p* < 0.001 with ANOVA, ns: no significant difference.

To detect the activity of cardiac STING in CVB3-induced VMC, we examined the STING-relevant molecules in the cardiac tissue of VMC mice. We found that CVB3 infection could increase the expressions of cardiac cGAS, STING, phosphor-TBK1, and phosphor-NF-κB/p65 proteins in CVB3-infected VMC mice (Figure 3A,B). In essence, these findings indicate that myocardial mtDNA might activate the STING-NF-kB signaling in cardiac macrophages to aggravate the VMC disease.

### 3.4. Myocardial mtDNA Drives the Macrophage Inflammatory Response in a STING-Dependent Manner

**Figure 4:**
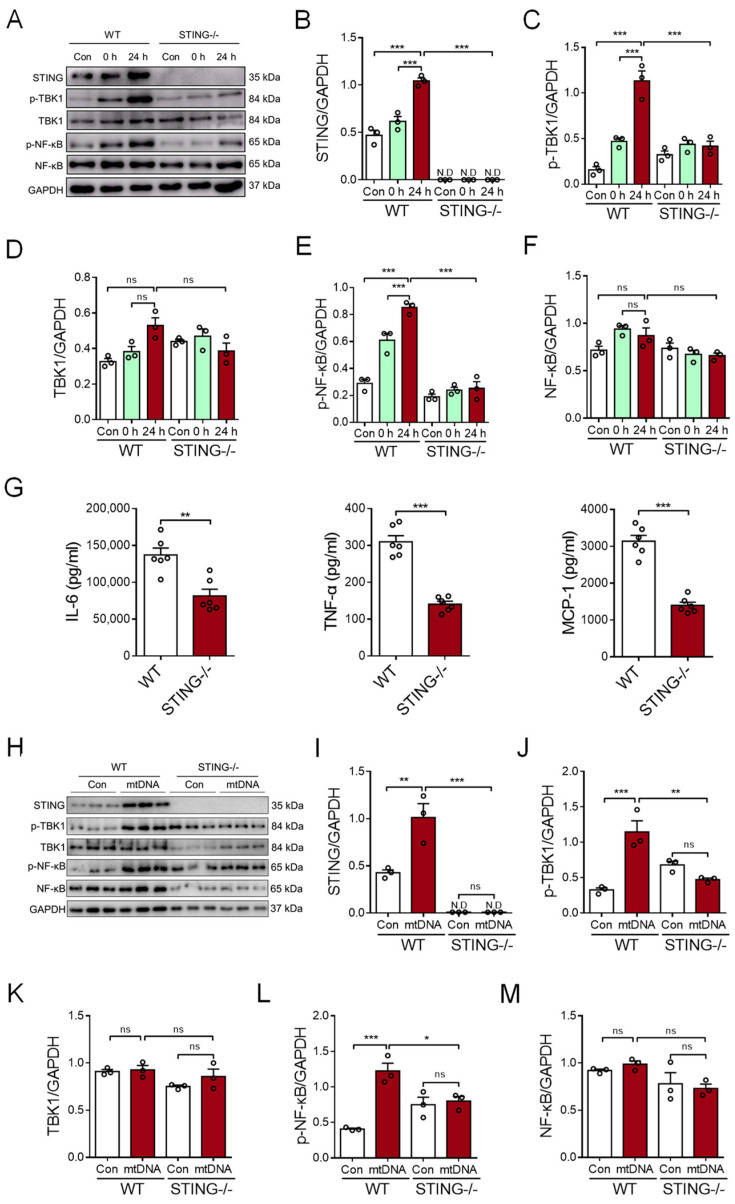
Myocardial mtDNA induces macrophage inflammatory responses in a STING-dependent manner. (**A**–**F**) Wild-type (WT) and *STING*-deficient BMDMs were cultured in a conditioned medium, using the CS from CVB3-infected cardiomyocytes, and STING activation was detected by analyzing the expressions of the indicated proteins. Mean ± SEM from three independent experiments in each group. (**G**) WT and *STING*-deficient BMDMs were cultured in a conditioned medium, using the CS from CVB3-infected cardiomyocytes, and inflammatory responses were detected. Mean ± SEM from six independent experiments in each group. (**H**–**M**) WT and *STING*-deficient BMDMs were cultured with or without mtDNA from CVB3-infected cardiomyocytes, and STING signaling was detected. Mean ± SEM from three independent experiments in each group. * *p* < 0.05, ** *p* < 0.01, *** *p* < 0.001 with ANOVA (**B**–**F**,**I**–**M**) and *t*-test (**G**). ns, no significant difference. N.D, not detected.

To determine the possible role of STING signaling in the myocardial mtDNA-induced macrophage inflammatory response, the CS from CVB3-infected cardiomyocytes was used to stimulate BMDMs; then, analyses of intracellular STING activation were conducted. We revealed that CS treatment resulted in the activation of STING signaling in BMDMs, as evidenced by the increased expression of phosphor-TBK1 and phosphor-NF-κB/p65 (Figure 4A–F). Moreover, *STING*-deficient BMDMs resisted the myocardial mtDNA-induced inflammatory response, as they showed impaired STING signaling (Figure 4A–F) and the production of inflammatory cytokines appeared diminished (Figure 4G).

To ensure the critical function of STING in the myocardial mtDNA-induced macrophage inflammatory response, mtDNA isolated from CVB3-infected cardiomyocytes was used to stimulate the *STING*-deficient and wild-type (WT) macrophages. Again, we observed that myocardial mtDNA efficiently activated STING signaling in WT macrophages, but not the *STING*-deficient counterparts (Figure 4H–M). In sum, CVB3 infection-induced myocardial mtDNA induces macrophage inflammation through the STING/NF-κB pathway.

### 3.5. STING Reflects the Disease Severity of CVB-Induced VMC

**Figure 5:**
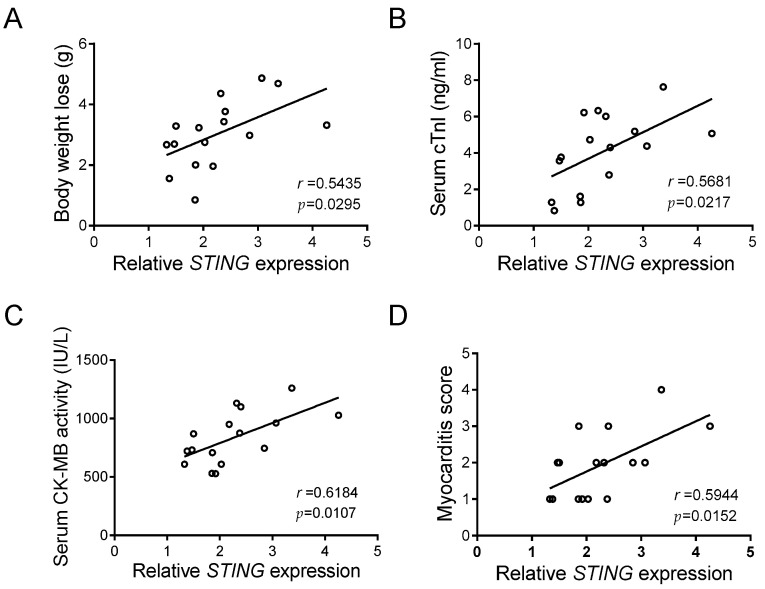
STING expression correlates with myocardial function in CVB3-infected mice. Correlation analyses of the myocardial mRNA level of STING and body weight loss (**A**), serum cTnI level (**B**), serum CK-MB activity (**C**), and myocarditis score (**D**) in CVB3-infected mice (*n* = 16). Pearson correlation analysis.

The above results pinpointed a crucial function of STING in macrophage inflammation caused by CVB3 infection, in that a noteworthy elevation of cardiac STING occurs in CVB3-induced VMC. Although body weight loss, serum cTnI, serum CK-MB activity, and the myocarditis score are reliable markers of VMC [30], we found that cardiac STING expression was positively and significantly associated with those indicators (Figure 5A–D), and that it closely reflects the disease severity of CVB3-induced VMC.

### 3.6. STING Deficiency Alleviates the Disease Development of CVB3-Induced VMC

To investigate the potential role of STING in the pathological processes of VMC, we analyzed the disease development of CVB3-induced VMC using STING knockout (STING-KO) and WT mice.

**Figure 6:**
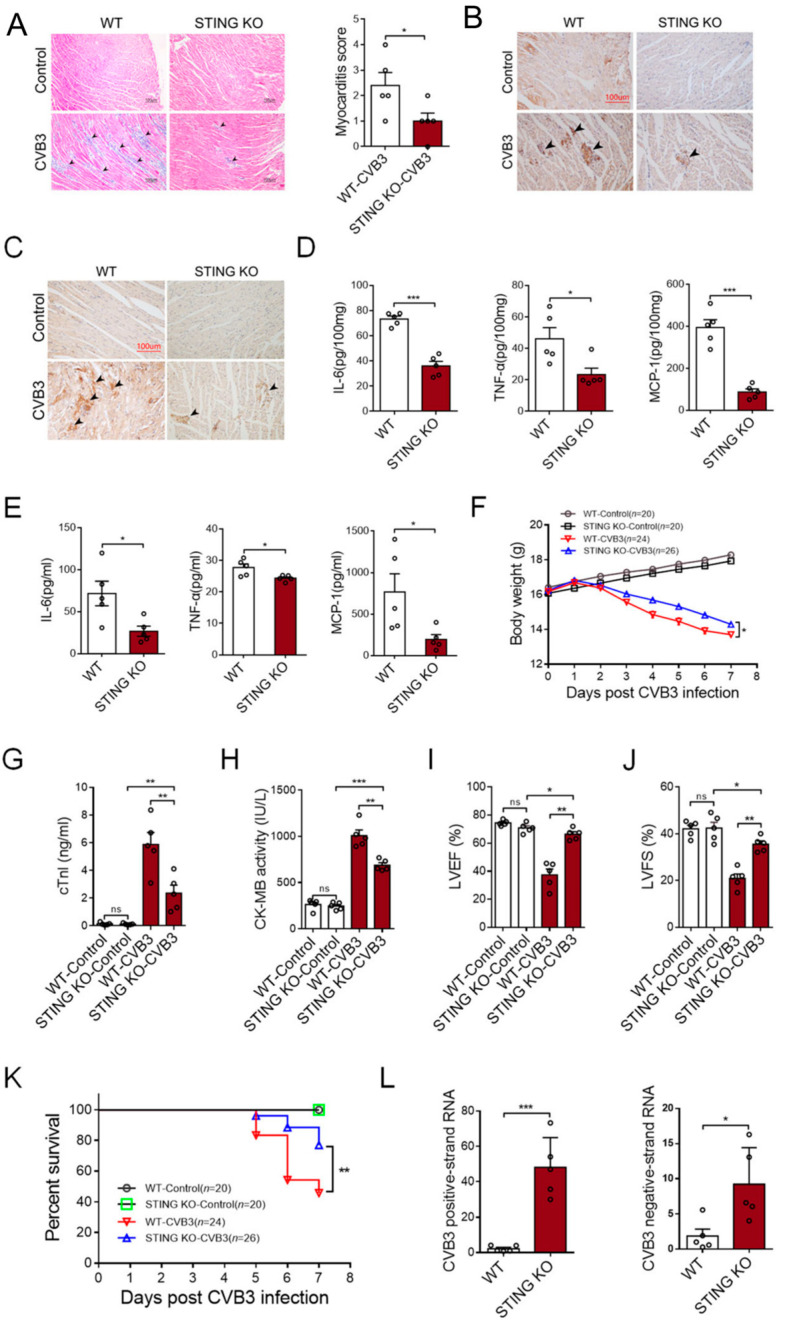
Deficiency of STING abrogates the development of CVB3-induced VMC. WT and *STING*-deficient mice were infected with CVB3 for 7 days, and the myocardial histology (100×, (**A**), black arrow: inflammation site, *n* = 5), myocardial CD45 (200×, (**B**), black arrow: CD45^+^ infiltrated leukocytes), F4/80 expression (200×, (**C**), black arrow: F4/80^+^ infiltrated macrophages), myocardial levels of the indicated pro-inflammatory cytokines ((**D**), *n* = 5), serum levels of the indicated pro-inflammatory cytokines ((**E**), *n* = 5), body weight loss ((**F**), *n* = 7), serum cTnI levels ((**G**), *n* = 5), serum CK-MB activity ((**H**), *n* = 5), myocardial function ((**I**,**J**), *n* = 5), survival rates (**K**), and CVB3 RNA (**L**) were analyzed. * *p* < 0.05, ** *p* < 0.01, *** *p* < 0.001 with *t*-test (**A**,**D**,**E**,**L**) and ANOVA (**G**–**J**). ns: no significant difference.

Compared with the WT mice, STING-KO mice showed ameliorated cardiac inflammation (Figure 6A), as well as a reduced number of CD45^+^ leukocytes (Figure 6B) and F4/80^+^ macrophages (Figure 6C). As a result, STING KO mice efficiently abrogated the generation of inflammatory cytokines in the cardiac tissues (Figure 6D) and peripheral blood (Figure 6E). Furthermore, STING-KO mice maintained body weight relatively well after CVB3 infection (Figure 6F), serum cTnI and CK-MB levels diminished (Figure 6G,H), as did functional cardiac capacities, evidenced by the elevated left ventricular ejection fraction (LVEF%) and fractional shortening (LVFS%) (Figure 6I,J). Accordingly, STING-KO mice exhibited higher survival rates in response to CVB3 infection (Figure 6K), although CVB3 replication might be enhanced in those mice (Figure 6L). These results indicate that STING could be a promising target for therapeutic options that work against CVB3-induced VMC.

## 4. Discussion

VMC is an inflammatory cardiovascular disease that typically results from a cardiotropic viral infection, and it causes the active inflammatory destruction of the myocardium [31,32]. Owing to the difficulties in diagnosis and treatment, VMC remains a major threat to human health. CVB3-induced VMC is an important class of VMC. Upon viral infection, CVB3 not only causes direct damage to the heart tissue, but it also triggers host–cell inflammatory responses [33]. Functionally, inflammation is considered a protective immune response, but evidence suggests that an excessive inflammatory response frequently produces harmful effects [34,35]. Accumulating evidence indicates that inflammation is the core driving factor of VMC [9]. However, the pathophysiological mechanisms of this complex disease remain largely unknown.

STING is a key adaptor protein, and its primary role is to trigger the innate immune response when it senses cytosolic dsDNA [36,37,38,39]. STING signaling activation mostly depends on cyclic GMP-AMP synthase (cGAS), a cytoplasmic DNA sensor [21,40]. In recent years, mounting evidence has shown that STING signaling is associated with a variety of diseases, particularly those linked to inflammation [21,41,42], including NASH [43], colitis [44], retina inflammation [45], and renal inflammation [46]. Herein, we showed that CVB3 infection can cause mtDNA to be released from cardiomyocytes. We proposed that injury to cardiac cells would lead to DNA leakage, and subsequent experiments confirmed our speculation. mtDNA was detected in the culture supernatant of CVB3-infected cardiomyocytes in vitro. Of note, myocardial mtDNA can be ingested by macrophages, and consequently, the transcription and secretion of inflammatory cytokines are enhanced in a STING-dependent manner. These results indicated that the mtDNA-STING cascade plays a critical role in the cardiac inflammation of CVB3-induced VMC.

In the current study, we revealed that the macrophage cGAS/STING/NF-κB signaling pathway could be activated by myocardial mtDNA, thus connecting the crosstalk between macrophages and cardiomyocytes in cardiac tissues upon CVB3 infection. Considering that macrophages were the primary infiltrating inflammatory cells, and that they play an important role in VMC [47,48], we believe they are likely to be the main contributors to CVB3-induced cardiac inflammation. Indeed, CVB3 infection induced a robust STING expression in myocardial macrophages, but not in cardiomyocytes.

STING primarily regulates inflammation by activating the NF-κB signaling pathway [49,50], which therefore plays a critical role in VMC [32]. Upon CVB3 infection, several RNA sensors, including TLR3 [10], TLR4 [51], and RIG-I/MDA5 [52], are activated to produce inflammatory responses. In contrast, we pinpointed the STING/NF-κB signaling pathway to be crucial in CVB3-induced VMC. Cardiac STING was elevated and activated by mtDNA released from damaged cardiomyocytes during CVB3 infection. Furthermore, CVB3 possesses adequate virulence to induce cardiomyocyte death via both the necrotic and apoptotic pathways [33,53].

Given the critical role of STING in the CVB3-induced macrophage inflammatory response, cardiac STING was positively correlated with the disease severity of CVB3-induced VMC. Of note, the knockout of STING efficiently abrogated the disease development of CVB3-induced VMC, thus improving the cardiac function and survival rates of the VMC models. Thus, targeting a classical DNA sensor STING could be helpful for protecting against an RNA viral infection. In accordance with our findings, STING has been reported to play an important role in cardiovascular disorders [54]. STING deficiency could attenuate myocardial inflammation and endoplasmic reticulum stress caused by aortic ligation [55]. The loss of STING also increases survival rate, reduces the expression of inflammatory cytokines and chemokines, reduces the cardiac infiltration of inflammatory cells, and improves heart function in mice with myocardial infarction [56].

**Figure 7:**
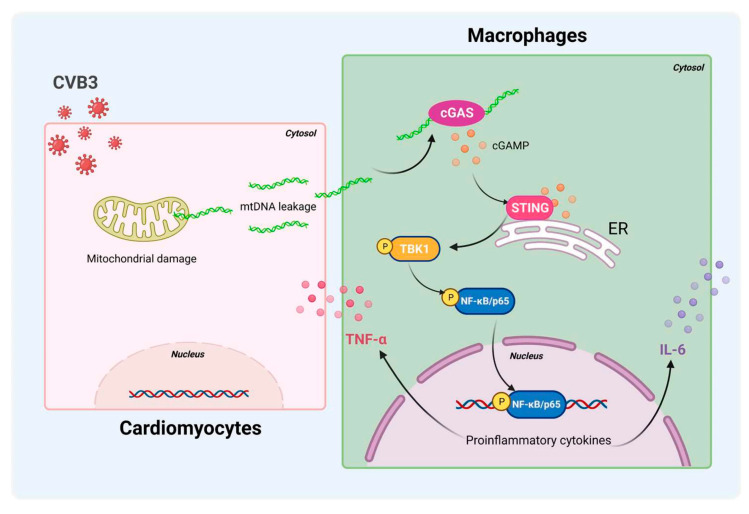
Schematic diagram of CVB3-induced myocardial inflammation. Upon CVB3 infection, cardiomyocytes release mtDNA into the extracellular myocardial environment, promoting cGAS/STING/NF-kB signaling in myocardial macrophages, and thus, inducing pro-inflammatory cytokines.

In summary, STING/NF-κB signaling in cardiac macrophages is activated by cardiomyocyte-derived mtDNA during CVB3 infection to a significant extent (Figure 7). Targeting STING could be a useful strategy to treat CVB3-induced VMC. These findings identify myocardial mtDNA as the crucial driver of cardiac inflammation, and cardiac macrophage STING as a novel regulator of the NF-κB inflammatory response; this provides new targets for therapeutic options that work against CVB3-induced VMC.

## Data Availability

All data supporting the findings were included in the current study.

## Supplementary Materials

The following supporting information can be downloaded at https://www.mdpi.com/article/10.3390/cells12212555/s1, Figure S1: Myocardial mtDNA induces macrophage inflammatory responses.
