# Supplementary material for: Myocardial Mitochondrial DNA Drives Macrophage Inflammatory Response through STING Signaling in Coxsackievirus B3-Induced Viral Myocarditis
Source: Cells. 2023 Oct 31;12(21):2555. doi: 10.3390/cells12212555 (PMC10648438; doi:10.3390/cells12212555)
Supplement: Supplementary file 1 [file cells-12-02555-s001.zip › cells-2592994-supplementary.pdf]

Supplementary Figure S1

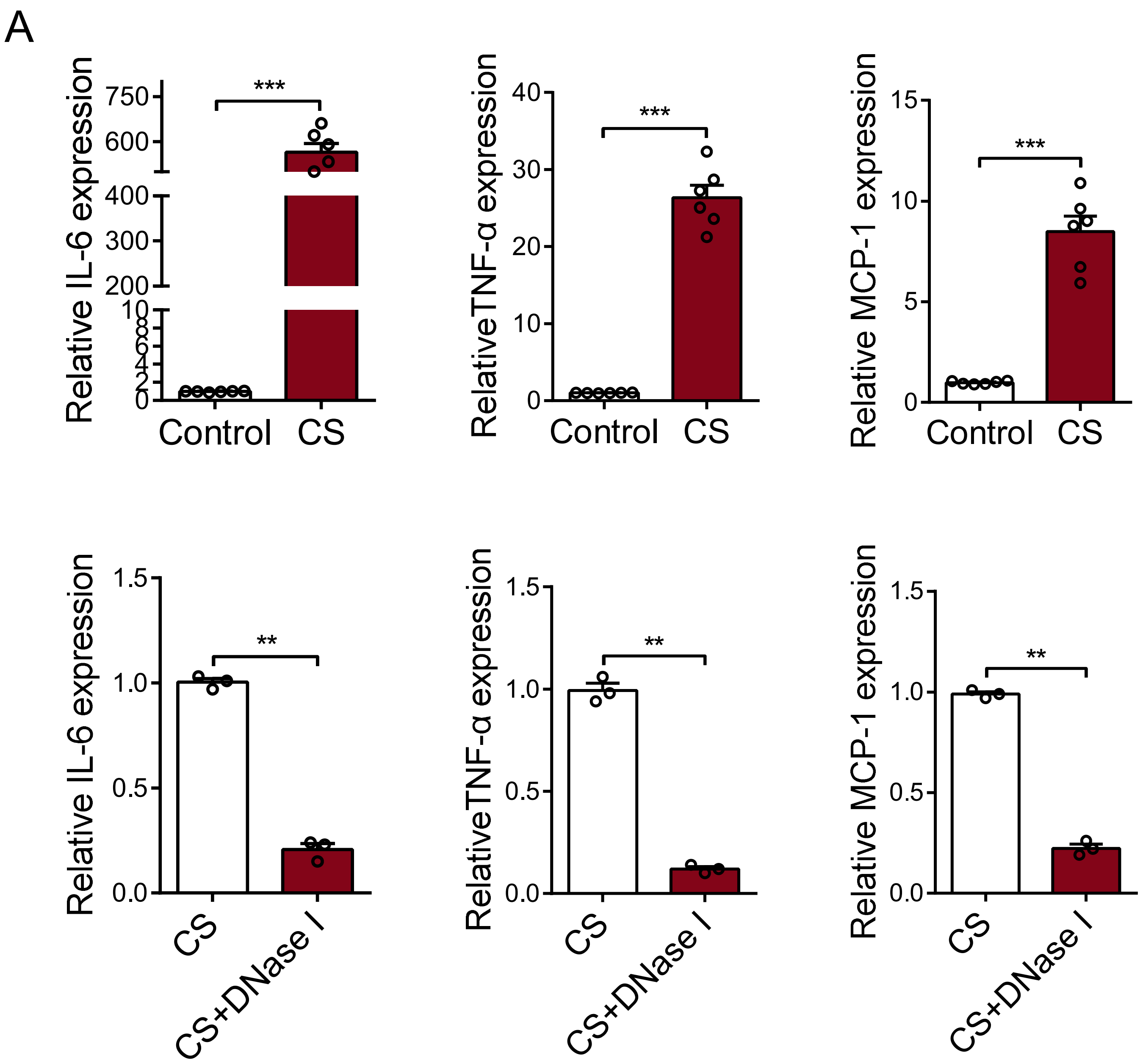

Figure S1. Myocardial mtDNA induces macrophage inflammatory responses (A) BMDMs were cultured with conditioned medium using culture supernatant (CS) from CVB3-infacted cardiomyocytes, and detected for IL-6, TNF-α, and MCP-1 expressions by qPCR. Mean ± SEM from 6 independent experiments in each group. (B) CS from CVB3-infected cardiomyocytes were pretreated with or without DNase I, and subsequently incubated with BMDMs for analyzing the inflammatory responses. Mean ± SEM from 3 independent experiments in each group. $^{**}p < 0.01$, $^{***}p < 0.001$ with *t-test*.
